# RSAD2-CMPK2 Signaling Mediates PVT1 Exon 9 Action in an AR-Independent Manner in PVT1 Exon 9 Overexpressing Neuroendocrine Prostate Cancers

**DOI:** 10.51894/001c.164007

**Published:** 2026-07-31

**Authors:** Rachel E. Bonacci, Seidu Adams, Chinedum C. Udekwu, Siti Khaula Nurazizah, Luke Washington, Olorunseun O. Ogunwobi

**Affiliations:** 1 College of Osteopathic Medicine, Michigan State University

## 09

### Introduction

Neuroendocrine prostate cancer (NEPC) is a deadly disease with poor response to conventional therapeutics. There is no FDA-approved molecular targeted treatment for NEPC, and the lack of therapeutics is reflected in the overall survival rate, which remains around 9 months. We have previously reported that PVT1 exon 9 overexpression induces the development of in vivo NEPC tumors.

### Objectives

We hypothesize that inhibition of PVT1 exon 9 is an effective therapeutic target in NEPC. Understanding the underlying molecular mechanisms that drive aggressive prostate cancer subtypes is desperately needed. Here, we report findings from the characterization of the novel PVT1 exon 9 overexpressing NEPC subtype.

### Methods

Western blotting, RT-qPCR, ELISA, xenograft modeling

### Results

Upregulation of PVT1 exon 9 leads to the overexpression of the innate immune surveillance proteins RSAD2 and CMPK2. PVT1 exon 9 directly binds to the RSAD2 protein as well as the CMPK2 protein. We have observed that CMPK2 overexpression is caused by the overexpression of RSAD2. Both RSAD2 and CMPK2 proteins do not directly interact, but the RSAD2 protein does bind to the CMPK2 transcript within the nucleus, leading to changes in CMPK2 RNA stability. CMPK2 overexpression is not involved in mediating interferon gamma signaling and is downstream of this signaling network. PVT1 exon 9 overexpression leads to androgen receptor (AR) suppression via an unknown mechanism independent of RSAD2 or CMPK2 activity. Targeting PVT1 exon 9, at its 5’ end, with an antisense oligonucleotide (ASO_ex9_2) leads to loss of PVT1 exon 9 expression, decoupling of PVT1 exon 9 from RSAD2 and CMPK2 proteins, re-expression of AR, and loss of tumor phenotypes in vitro and in vivo. Co-treatment with ASO_ex9_2 and the AR inhibitor enzalutamide leads to significant inhibition of NEPC tumor phenotypes in vitro and in vivo.

### Discussion

This novel signaling mechanism present in a subset of NEPC is a clinically relevant and targetable molecular aberration. Further investigation is warranted to understand the best treatment strategy for this subtype, as well as identify other aggressive prostate cancer subtypes that may benefit from PVT1 exon 9 inhibition.

